# Tailoring the dispersion characteristics in planar arrays of discrete and coalesced split ring resonators

**DOI:** 10.1038/s41598-023-47216-3

**Published:** 2023-11-15

**Authors:** Ioannis Spanos, Christopher John Stevens, Laszlo Solymar, Ekaterina Shamonina

**Affiliations:** Department of Engineering Science, University of Oxford, Parks Road, Oxford, OX1 3PJ UK

**Keywords:** Electrical and electronic engineering, Condensed-matter physics

## Abstract

In this report, the coupling and dispersion characteristics of discrete and coalesced square resonators was investigated in the MHz regime. Resonators with one and three gaps were considered. When the resonators are not in direct contact, the number of gaps has little effect upon the total coupling, which is negative. When the resonators are connected so that they share one side, the coupling can change drastically depending on the number of gaps. In particular, when the shared side has a gap, the total coupling coefficient switches to positive values, making it possible for forward travelling waves to propagate on arrays. Experimental, numerical and analytical data verify this behaviour.

## Introduction

Split ring resonators (SRRs) have attracted significant research interest throughout the years, owning to their strong magnetic response^1^. In a single SRR a circulating current will create a magnetic field normal to the plane of the resonator. Two SRRs positioned close enough can communicate with their magnetic fields; a time-varying current in one, induces a current in the second via the law of induction. In an array of SRRs, the induced currents will propagate along the line like waves. Since the resonators are magnetically coupled to each other, these waves have been given the name magnetoinductive (MI) waves^2,3^. MI waves have found application in imaging^4,5^, sensing^6,7^, conductivity detection^8^, wireless power transfer^9,10^ and waveguides^11,12^, among others. If the elements are electrically coupled, a similar behaviour is observed. In this case the waves are called electroinductive (EI) waves^13–15^.

A SRR can be modelled by a circuit equivalent, having a self-inductance from the conductive part and a capacitance due to the gap. The shape of the SRR can differ, but the response remains similar^16–18^. The element’s resonance can then be described by this circuit. This approximation is quite effective when the size of the SRR is smaller than the wavelength at resonance^19–21^. Hence, a medium made of coupled SRRs can be modelled analytically^2,22^. Both backward- and forward-travelling MI waves can be controlled via the coupling between the elements. Typically, axial arrays result in forward-wave propagation because the elements have a positive coupling coefficient, while planar arrays show backward-wave propagation because the elements have a negative coupling coefficient. Furthermore, the relative orientation between SRRs can affect the coupling mechanisms. Depending on how close the gaps of neighbouring SRRs are, the electric coupling can increase in magnitude^23^. For the same reason, it can be expected that the number of gaps will also affect the coupling^24^. The coupling is affected by the frequency as well. In the THz regime, the magnetic coupling weakens significantly, resulting in the electric coupling being dominant^25,26^. At the same time, the coupling coefficients can become complex, altering the shape of the band due to retardation effects^12^. For these reasons, it is crucial that the coupling coefficients can be reliably measured. An analytical method of measuring the complex magnetic and electric coupling coefficients from experimental or numerical data was proposed in^27^. Understanding how the couplings change is beneficial. In^28^ the resonators of a metasurface are carefully optimized using a developed analytical model so that their electric and magnetic responses are balanced. The metasurface is shown to be capable of manipulating transmitted wave fronts while exhibiting high transparency over a broad range of frequencies was demonstrated.

Even though the magnetic and electric coupling between discrete elements has been extensively studied, reports on coalesced resonators have been limited. There are a few examples where coalesced SRRs for applications at optical frequencies are investigated^29–31^; however, the effect on the total coupling is not yet understood. The term conductive coupling was coined to describe the change in coupling due to the elements touching. In the analysis that follows it will be shown that the magnetic and electric couplings are sufficient to describe the behaviour of coalesced resonators, without the need to introduce additional coupling mechanisms.

Coalesced elements could lead to some interesting results. It has been observed that a planar array can support the propagation of forward-waves if the SRRs are joined^32^. In this case, the coupling between elements appears to turn positive when the SRRs are coalesced. A similar effect can be observed when the SRRs are connected via capacitive links, forming alternating major and minor loops^33,34^. This essentially creates a diatomic chain of resonators, making it possible to tailor the dispersion depending on the capacitance between the two types of loops. Our results show that the dispersion can be also tailored for backward and forward waves in a monoatomic chain.

Here, the coupling and dispersion of discrete and coalesced SRRs are investigated. Elements with 1–4 gaps have been considered. However, the results for the two- and four-gap cases are not shown because they are not sufficiently different. The analysis that follows will be restricted to the one- and three-gap cases. Analytical results are compared with experimental and numerical data for each case. In "Analytical theory" section, the analytical model is constructed. "Experimental and numerical setup" section gives a summary of the experimental and numerical setups. In Sect. "Results" section, results for the unit cell, dimer and array samples are presented. Finally, the report ends with the conclusions in "Discussion" section".

## Analytical theory

A SRR with one gap can be viewed as a RLC circuit, where *R* is the resistance, *L* is the self-inductance of the resonator and *C* is the capacitance at the gap. Doing so, the element’s resonance frequency and quality factor can be expressed as

1 $$\begin{aligned} f_0=\frac{\omega _0}{2\pi }=\frac{1}{2\pi }\sqrt{\frac{1}{LC}} \hspace{1cm} \text {and} \hspace{1cm} Q=\frac{\omega _{0}L}{R}\,. \end{aligned}$$

**Figure 1:**
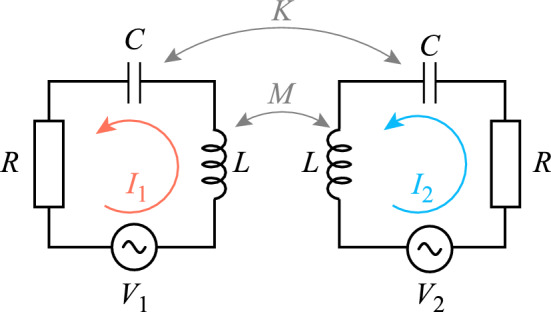
Equivalent circuit model for a two-element system. Each SRR has a resistance *R*, self-inductance *L* and capacitance *C*. There is a mutual inductance *M* and mutual capacitance *K* between the two elements. $I_{1,2}$ are the currents of elements 1 and 2, while $V_{1,2}$ are the applied voltages to elements 1 and 2.

A two-element system where the SRRs are both magnetically and electrically coupled (Fig. 1) can be described using Kirchoff’s equations as

2 $$\begin{aligned} &Z_0I_1+j\omega MI_2+\frac{1}{j\omega K}I_2=V_1 \\&Z_0I_2+j\omega MI_1+\frac{1}{j\omega K}I_1=V_2 \\&Z_0=R+j\omega L+\frac{1}{j\omega C}=j\omega L\left( 1-\frac{\omega _0^2}{\omega ^2}-j\frac{\omega _0}{Q\omega }\right) . \end{aligned}$$

**Figure 2:**
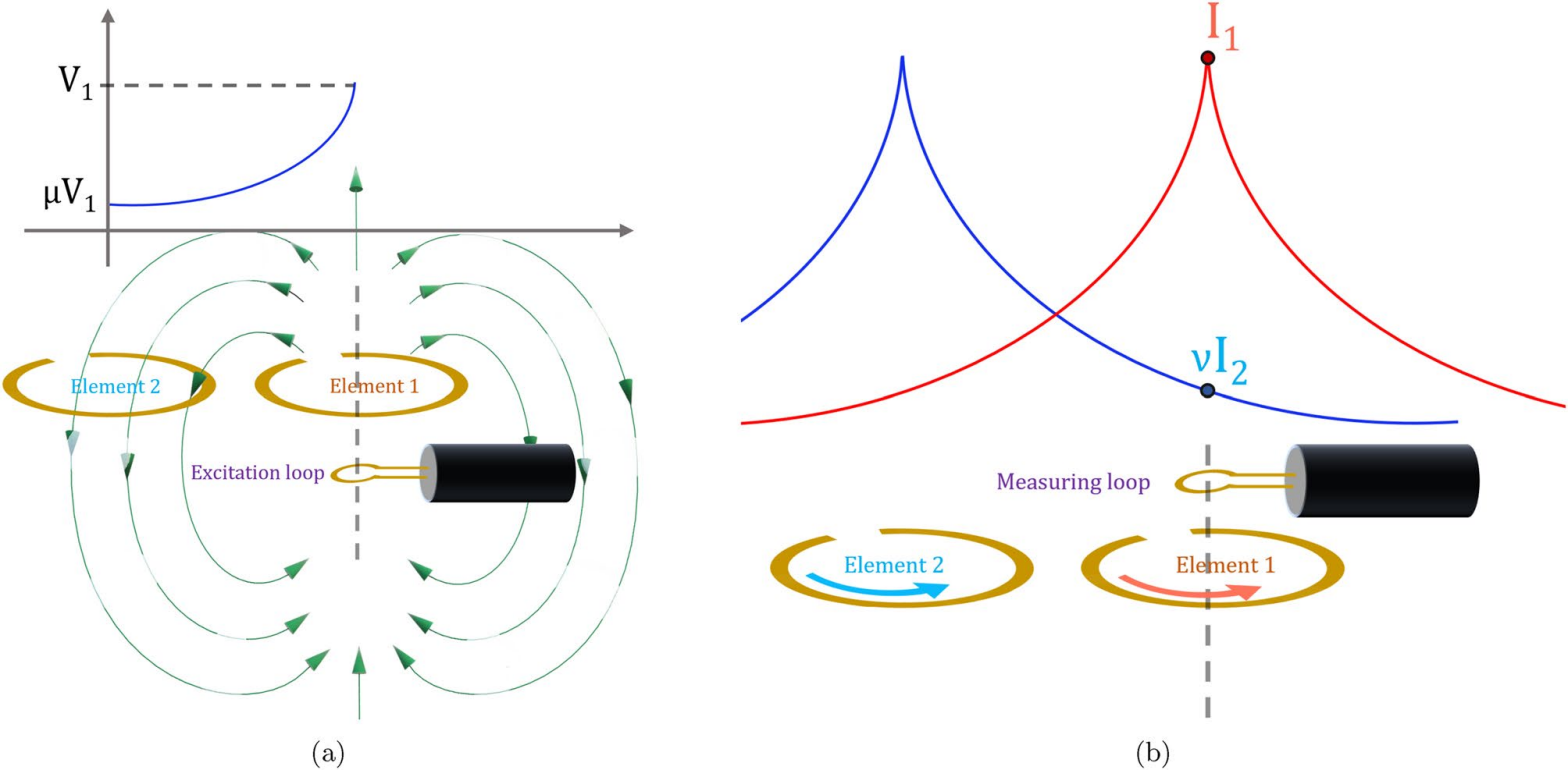
Additional interaction between the excitation/measuring loops and the SRRs. (**a**) Correction factor $\mu$ due to the excitation loop’s magnetic field reaching element 2, and (**b**) Correction factor $\nu$ due to the measuring loop picking the magnetic field of element 2.

Here $Z_0$ is the impedance of a single SRR, $I_{1,2}$ are the currents of elements 1 and 2, *M* and *K* are the mutual inductance and capacitance, while $V_{1,2}$ are the applied voltages to elements 1 and 2 respectively. In the case that only element 1 is excited $V_2$ should be zero. However, when a magnetic loop is used to induce a voltage on element 1, some of its magnetic field will also reach element 2, resulting in $V_2\ne 0$ (Fig. 2a). At the same time, a magnetic loop placed above element 1, $H_1$, will mainly record the magnetic field produced by the current of element 1 but will also pick up some of the magnetic field due to the current of element 2 and vice versa (Fig. 2b), so that

3 $$\begin{aligned}{}&V_2=\mu V_1 \\&H_1 \rightarrow I_1+\nu I_2 \\&H_2 \rightarrow \nu I_1+I_2\,, \end{aligned}$$

where $\mu$ and $\nu$ are corrections factors accounting for the unwanted interaction of the loops and the SRRs^27^. Their symbols have been changed from $\alpha$ and $\beta$ to avoid confusion with the dispersion diagrams later on. The correction factors can be calculated by removing one of the two SRRs from the setup. In the following, superscript *F* symbolizes that only the first element was present during the measurement and superscript *S* shows that only the second element was present during the measurement, so that

4 $$\begin{aligned} \mu =\frac{H_2^S}{H_1^F} \hspace{1cm} \text {and} \hspace{1cm} \nu =\frac{H_1^S}{H_2^S}=\frac{H_2^F}{H_1^F}\,. \end{aligned}$$

The actual currents $I_{1,2}$ can be calculated from Eq. (3)

5 $$\begin{aligned} I_1=\frac{H_1-\nu H_2}{1-\nu ^2} \hspace{1cm} \text {and} \hspace{1cm} I_2=\frac{H_2-\nu H_1}{1-\nu ^2}\,. \end{aligned}$$

If the magnetic and electric coupling coefficients are assumed as

6 $$\begin{aligned} \kappa _H=\frac{2M}{L} \hspace{1cm} \text {and} \hspace{1cm} \kappa _E=\frac{2C}{K}\,, \end{aligned}$$

then Eq. (2) can be rearranged to

7 $$\begin{aligned} \kappa _H-\frac{\omega _0^2}{\omega ^2}\kappa _E=-2\frac{\displaystyle \frac{I_2}{I_1}-\mu }{1-\mu \displaystyle \frac{I_2}{I_1}}\left( 1-\frac{\omega _0^2}{\omega ^2}-j\frac{\omega _0}{\omega Q}\right) , \end{aligned}$$

where the ratio of currents is calculated from Eq. (5). If the right side of Eq. (7) is calculated and plotted versus $\omega _0^2/\omega ^2$ (or $f_0^2/f^2$) the graph should be of a line, whose intercept and slope are equal to $\kappa _H$ and $-\kappa _E$ respectively. Furthermore, the real and imaginary parts of the coupling coefficients can be calculated separately, which is important in the presence of retardation. Similarly to the two-element system and assuming only first neighbour interactions, in an one dimensional array of *N* resonators, Kirchoff’s equation for the $m_{th}$ element is

8 $$\begin{aligned} Z_0I_m+\left( j\omega M+\frac{1}{j\omega K}\right) (I_{m-1}+I_{m+1})=0\,. \end{aligned}$$

In a periodic structure, wave propagation can be assumed

$$\begin{aligned} I_m=I_0e^{-jmkd}\,, \end{aligned}$$

where $I_0$ is the wave’s amplitude, $k=\beta -j\alpha$ is the wave number with $\beta$ and $\alpha$ being the dispersion and attenuation respectively, and *d* is the unit cell size. The currents of any three consecutive elements $I_{m-1}$, $I_m$, $I_{m+1}$ are connected through trigonometry as

9 $$\begin{aligned} I_{m-1}+I_{m+1}=2I_m\cos {kd}\,. \end{aligned}$$

This way, Eq. (8) is transformed into

10 $$\begin{aligned} \cos {kd}=\cos {(\beta d-j\alpha d)}=-\frac{1-\displaystyle \frac{\omega _0^2}{\omega ^2}-j\frac{\omega _0}{Q\omega }}{\left( \kappa _H-\displaystyle \frac{\omega _0^2}{\omega ^2}\kappa _E\right) }\,. \end{aligned}$$

The dispersion and attenuation curves can be plotted analytically through Eq. (10), by knowing the resonance, quality factor and coupling coefficients, and through Eq. (9) by using experimental or numerical data of each resonator’s currents.

## Experimental and numerical setup

**Figure 3:**
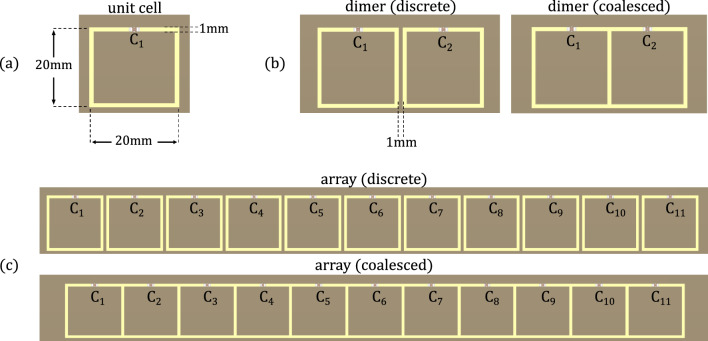
The one-gap group of PCB samples: (**a**) The unit cell, (**b**) Discrete and coalesced dimers, and (**c**) Discrete and coalesced 11-element arrays. Images from KiCad.

Two groups of square resonators were fabricated and measured: one-gap and three-gap. The one-gap samples are shown in Fig. 3. All resonators are squares of side 20 mm, track width 1 mm and gap size 1 mm. The spacing between them is either 1 mm (discrete) or −1 mm (coalesced). Each group consists of a unit cell (for measuring $f_0$, *Q*, correction factors), two dimers (for calculating $\kappa _H$, $\kappa _E$ for 1 mm and −1 mm spacing) and two 11-element arrays (for extracting dispersion, attenuation for 1 mm and −1 mm spacing). The samples were fabricated on single-layered printed circuit boards (PCBs) with FR4 TG130−140 as the base material. The copper tracks have a thickness of $35\,\upmu \hbox {m}$. The PCB dimensions are 100 mm $\times$ 100 mm for the unit cell and dimers, and 270 mm $\times$ 100 mm for the 11-element arrays, while the dielectric substrate’s thickness in all samples is 1.6 mm. The solder resist layer around the resonators was removed in the design stage, so that capacitors could be soldered at the gaps. On their own, the resonators have a resonance in the range of 2–4 GHz depending on the number of gaps. This could be problematic because the wavelength is no longer significantly larger than the unit cell size, an assumption on which the analytical theory is founded on. Hence, 100 pF capacitors were soldered at the gaps of the resonators, to drive down the resonance to the MHz regime, where circuit theory can be safely used. All capacitors were individually measured prior to soldering, so that the ones with a tolerance of less than $1\%$ are used.

The measurements were performed using two magnetic loop antennas connected to a vector network analyzer (VNA), as the ones shown in Fig. 2. The loops had a diameter of 2.5 mm, wire thickness 1 mm and a gap size 1 mm. Element 1 would get excited by the first probe placed 2 mm below its center, while the second probe would move 4 mm above the center of each element to record the $S_{21}$ parameter. Background measurements without the samples were also recorded and subtracted from the measured $S_{21}$ during analysis.

The frequency domain solver of Computer Simulation Technology Microwave Studio (CST) was used for the numerical simulations. The geometries of Fig. 3 for the one-gap and three-gap cases were designed with the same dimensions as the PCB samples. The boundary conditions in all directions are set to open, as they would be in an experimental measurement. However, the bounding box size is a parameter that needs to be optimized, since having an infinitely big bounding box is not very efficient. During the simulation, a mesh of the structures inside the bounding box is created by dividing them into smaller finite elements. CST solves Maxwell’s equation for each element and combines them all together afterwards to approximate a solution for the larger structure. Naturally, the size of the mesh will affect the final result significantly. The simulation can be fitted to the experimental data, by choosing a combination of mesh and bounding box size that matches the measured $f_0$ and *Q* of the unit cell samples.

## Results

### Single resonator

**Figure 4:**
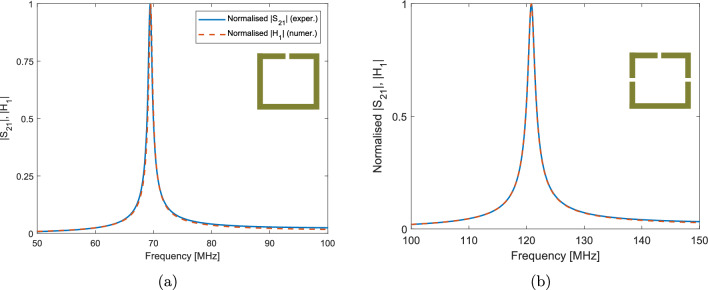
Normalised experimental $|S_{21}|$ and numerical $|H_1|$ measurements for the (**a**) One-gap, and (**b**) Three-gap unit cells.

The experimental $|S_{21}|$ parameter and numerical $|H_1|$ field for the unit cell samples are shown in Fig. 4. The mesh and bounding box were fitted to each resonator’s data. The values for $f_0$ and *Q* are shown in Table 1. As more gaps are introduced, the self-inductance of the resonator is reduced slightly, resulting in a 1–2 % deviation from the $\sqrt{n}$ dependence which is predicted for $f_0$ from Eq. (1).

**Table 1:** Resonance results.

Resonator	Resonant frequency, $f_0$ [MHz]	Quality factor, *Q*
1-gap	69.4	116
3-gap	121	105

### Dimers

**Figure 5:**
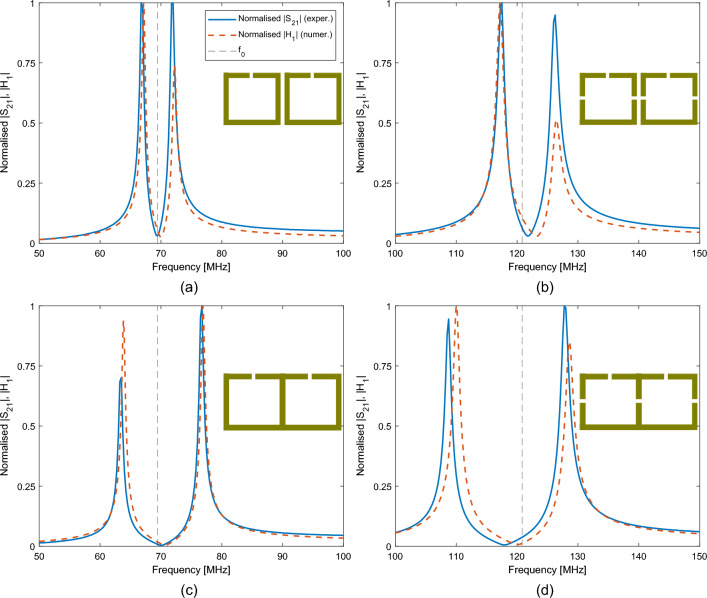
Normalised experimental $|S_{21}|$ and numerical $|H_1|$ measurements for the dimer samples. (**a**) and (**b**) Discrete case, (**c**) and (**d**) Coalesced case.

There are four dimer samples. The normalised experimental $|S_{21}|$ parameter and numerical $|H_1|$ field of the first resonator are shown in Fig. 5. The resonance of the unit cell is included as the dashed grey line. The agreement between experiment and simulation appears to slightly falter. The most likely reason is that CST produces an approximation for each geometry by creating a mesh. Although the simulation was fitted to the experimental data of the unit cell, when a second element is added, the geometry is changed and so is the meshing. Despite this, the agreement remains good overall. There is a resonance split around the original $f_0$, resulting in two peaks. The separation and the position of the two peaks compared to $f_0$, is a measure of the total coupling’s magnitude and sign. For example, the coalesced cases appear to have a larger coupling compared to their discrete counterparts. Furthermore, in Fig. 5d the resonance split expands more towards lower frequencies, which is a sign of a positive coupling coefficient.

**Figure 6:**
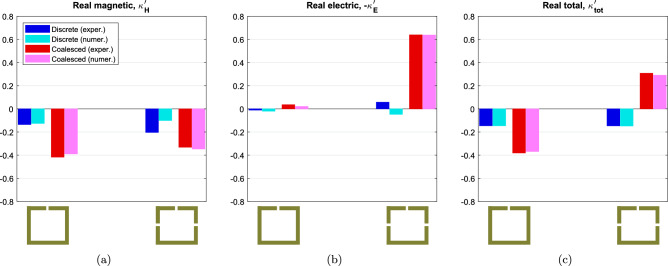
Real parts of the (**a**) Magnetic, (**b**) Electric and (**c**) Total coupling coefficients for the discrete and coalesced one-gap and three-gap resonators. The values of discrete resonators are shown with blue (experimental) and cyan (numerical), while the values of coalesced resonators are shown with red (experimental) and pink (numerical).

Since the experimental and simulation data are still in a good agreement, the combination of mesh and bounding box is kept the same. The correction factors from Eq. (4) were calculated as $\mu =-0.07$ and $\nu =-0.10$ for both experiment and simulation. Using the correction factors and the data of the dimer samples, the coupling coefficients can be extracted from Eq. (7) for both experiment and simulation. The calculated real parts of the coupling coefficients are shown in Fig. 6. The values of discrete resonators are shown with blue (experimental) and cyan (numerical), while the values of coalesced resonators are shown with red (experimental) and purple (numerical). One immediately notices the behaviour of $\kappa _{tot}^{'}$ in Fig. 6c, which was calculated by adding $\kappa _{H}^{'}$ and $-\kappa _{E}^{'}$. When the resonators are not in contact, $\kappa _{tot}^{'}$ is negative and constant, regardless of the number of gaps. It can be seen in Fig. 6a and b that the main contribution comes from the magnetic coupling coefficient $\kappa _{H}^{'}$, while $\kappa _{E}^{'}$ is near zero for both the experimental and numerical setup. Even though there are minor disagreements between the two, the total coupling $\kappa _{tot}^{'}$ values are almost identical. When the resonators are coalesced, $\kappa _{H}^{'}$ increases in magnitude and $\kappa _{E}^{'}$ stays roughly the same for the one-gap case. However, in the three-gap case where the shared side has a gap, $\kappa _{E}^{'}$ has a dramatic increase resulting in $\kappa _{tot}^{'}$ to turn positive. The agreement between experimental and numerical results is excellent. This switch to positive values means that a planar array will be able to support the propagation of forward travelling waves. The imaginary parts of the coupling coefficients were calculated less than $<1\%$ of their real counterparts and are not shown. The presence of retardation should be limited in the dispersion curves.

### Arrays

To verify the above results, the 11-element arrays were analyzed. First, the dispersion and attenuation curves are calculated analytically through Eq. (10). At the same time, assuming that for resonator *m*

$$\begin{aligned} I_m \sim H_m \sim S_{21}^m\,, \end{aligned}$$

**Figure 7:**
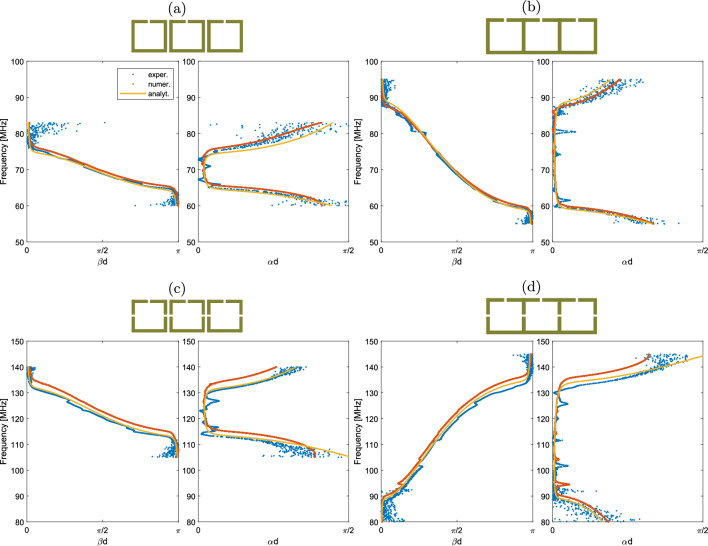
Dispersion and attenuation for the (**a**,**b**) One-gap, and (**c**,**d**) Three-gap cases. The experimental and simulation data were calculated from the currents of three consecutive elements. The analytic model includes losses and retardation.

Equation (9) can be used to plot the dispersion and attenuation curves from the experimental and numerical measurements of three consecutive resonators. Even though Eq. (9) is true for an infinite periodic structure, it can be used as an approximation on the 11-element array because its two ends are sufficiently apart. This method of extracting the dispersion relies on the magnetic field of each resonator. In all cases, the frequencies outside the passband have relatively low magnetic fields, making the extracted dispersion unreliable in these parts, which have been removed. The experimental and numerical results, along with the analytical curves are shown in Fig. 7. For the analytical curve, the experimental $f_0$, *Q*, $\kappa _E$ and $\kappa _H$ were used. The blue dots are from experimental data, the red dots are from numerical data while the yellow line is from the analytical theory.

Overall, there is a good agreement between the three. There is backward wave propagation in all setups, except in the three-gap coalesced case where the $\kappa _{tot}^{'}$ becomes positive. The analytical model using the two coupling coefficients $\kappa _{H}$ and $\kappa _{E}$ is in excellent agreement with the experimental data, accurately predicting the switch from a backward to a forward travelling MI wave. As discussed previously, CST produces an approximate solution, resulting in a slight divergence from the experimental curves, which is not surprising given the frequency mismatch already arising in the dimer setups. The fractional normalised bandwidth is roughly $14\%$ for the discrete arrays, while it is close to $40\%$ for the coalesced arrays, due to the higher coupling between elements.

Square resonators with two and four gaps were investigated as well (not shown here). The results on one-gap and three-gap resonators were selected because they summarize the behaviour of discrete and coalesced resonators to the point: When resonators are discrete, the total coupling is negative and is not affected by the number of gaps. Magnetic coupling is the main contributor, while electric coupling is effectively zero. The structure can support the propagation of backward travelling waves. In the coalesced case, the number of gaps and whether the shared side is capacitively loaded, has a clear effect on the total coupling. This change is due to the electric coupling and not the magnetic coupling, which is still constant regardless of the number of gaps. If the shared side is capacitively loaded, the structure can support the propagation of forward travelling waves.

Assuming that the number of gaps in one resonator is *n*, the total capacitance per unit cell is

$$\begin{aligned} C=\frac{C_0}{n}\,, \end{aligned}$$

where $C_0$ is the capacitance of one gap. Using Eq. (6) and the extracted values of $\kappa _{E}$, the mutual capacitance is calculated as $K\approx -C_0$ across all coalesced resonators with a gap on their shared side. The electric coupling coefficient can then be shown to vary as

$$\begin{aligned} \kappa _E=\frac{2C}{K}\approx -\frac{2}{n}. \end{aligned}$$

So the coupling, and by extension the bandwidth, can be tailored by the number of gaps per unit cell, when the shared side of coalesced resonators is capacitely loaded.

## Discussion

The coupling of discrete and coalesced resonators in the MHz regime was investigated. It was shown that the magnetic and electric couplings are sufficient to describe the behaviour of coalesced resonators, without the need to introduce additional coupling terms due to the shared sides between neighbouring elements. It was observed that, the total coupling coefficient can be switched to positive values. This switching behaviour occurs when two conditions are met: the resonators are joined and their shared side has a gap. In the discrete cases, the number of gaps has little effect upon the coupling, with the magnetic coupling coefficient being negative and the electric coupling coefficient near zero. The one-gap coalesced case is similar, but the magnetic coupling increases in magnitude. In the three-gap coalesced case the electric coupling increases dramatically, resulting in the total coupling switching to positive values. This is confirmed by the dispersion curves, predicting forward wave propagation. With the addition of the loaded gap in the shared sides of the cells,our circuit resembles a model transmission line topology. In fact the system here is not the same, as our structures are open, resulting in significant coupling between adjacent cells via magnetic and electric interactions which are not normally considered in a transmission line model. The imaginary parts of the coupling coefficients remain small overall so that retardation has a minor effect on the band shape. At higher frequencies, the imaginary parts, and by extension retardation, are expected to have a greater effect on the band shape.

Equation (7) proved to be a reliable tool in extracting the complex coupling coefficients, which were used in plotting the analytical dispersion and attenuation curves, showing excellent agreement with the experimental data. Furthermore, the CST simulations can be fitted to the experimental data, by changing the mesh and bounding box size. Apart from a good agreement, this also allows for the calculation of the self inductance and the losses in each case. Even though the agreement between experiment and simulations slightly falters when more elements are added, the key features of the results are still there. So, if the simulation is fitted to the experimental data of a single element, the behaviour of structures with more elements could be reliably predicted.

In two dimensions the potential for coalescing along one or more axes offers a new degree of flexibility to the designer. For example a structure where coupling was positive in the x-axis and negative in the y-axis could be designed^35,36^. Such a mesh would support forward waves in the x direction and backward waves in the y direction whilst still being essentially planar. Even though this coupling situation can also be realized by stacking SRRs axially in the x-direction and placing stacks next to one another in the y-direction, the coalesced mesh will always be thin whilst the stacked SRRs are not.

## Data Availability

The data supporting the findings of this paper are available from the corresponding author upon reasonable request.
